# A novel quantitative computer-assisted drug-induced liver injury causality assessment tool (DILI-CAT)

**DOI:** 10.1371/journal.pone.0271304

**Published:** 2022-09-29

**Authors:** Hans L. Tillmann, Ayako Suzuki, Michael Merz, Richard Hermann, Don C. Rockey

**Affiliations:** 1 Division of Gastroenterology, Hepatology & Nutrition, East Carolina University, Greenville, NC, United States of America; 2 Greenville VA Health Care Center, Greenville, NC, United States of America; 3 Duke University Medical Center, Durham, NC, United States of America; 4 Durham VA Medical Center, Durham, NC, United States of America; 5 AstraZeneca, independent consultant, Freiburg, Germany; 6 AstraZeneca, Gaithersburg, MD, United States of America; 7 Digestive Disease Research Center, Medical University South Carolina, Charleston, SC, United States of America; Medizinische Fakultat der RWTH Aachen, GERMANY

## Abstract

**Background and aims:**

We hypothesized that a drug’s clinical signature (or phenotype) of liver injury can be assessed and used to quantitatively develop a computer-assisted DILI causality assessment-tool (DILI-CAT). Therefore, we evaluated drug-specific DILI-phenotypes for amoxicillin-clavulanate (AMX/CLA), cefazolin, cyproterone, and *Polygonum multiflorum* using data from published case series, to develop DILI-CAT scores for each drug.

**Methods:**

Drug specific phenotypes were made up of the following three clinical features: (1) latency, (2) R-value, and (3) AST/ALT ratio. A point allocation system was developed with points allocated depending on the variance from the norm (or “core”) for the 3 variables in published datasets.

**Results:**

The four drugs had significantly different phenotypes based on latency, R-value, and AST/ALT ratio. The median cyproterone latency was 150 days versus < 43 days for the other three drugs (median: 26 for AMX/CLA, 20 for cefazolin, and 20 for *Polygonum multiflorum*; p<0.001). The R-value for the four drugs was also significantly different among drugs (cyproterone [median 12.4] and *Polygonum multiflorum* [median 10.9]) from AMX/CLA [median 1.44] and cefazolin [median 1.57; p<0.001]). DILI-CAT scores effectively separated cyproterone and *Polygonum multiflorum* from AMX/CLA and cefazolin, respectively (p<0.001). As expected, because of phenotypic overlap, AMX/CLA and cefazolin could not be well differentiated.

**Conclusions:**

DILI-CAT is a data-driven, diagnostic tool built to define drug-specific phenotypes for DILI adjudication. The data provide proof of principle that a drug-specific, data-driven causality assessment tool can be developed for different drugs and raise the possibility that such a process could enhance causality assessment methods.

## Introduction

Drug-induced liver injury (DILI) is an important cause of acute liver injury and liver-related morbidity and mortality [1–4]. DILI is also a major concern in drug development and post-marketing surveillance, as evidenced by hepatotoxicity being a leading cause for market withdrawal of licensed drugs [5, 6]. Moreover, DILI diagnosis is extremely challenging, as liver biochemistry abnormalities may be occurring even in absence of clear cause of liver injury [7–9].

Unlike diseases such as viral hepatitis, where diagnostic testing may confirm or exclude the diagnosis with high sensitivity and specificity, DILI is a diagnosis based on clinical suspicion and exclusion of other causes of liver diseases. A variety of causality assessment methods (CAMs) have been developed and often use point-scoring systems (i.e., Roussel Uclaf Causality Assessment Method [RUCAM], “clinical diagnostic scale” [CDS]) [10–12]. Interestingly, these systems typically use slightly different algorithms for hepatocellular vs. mixed or cholestatic liver injury pattern. This approach is not borne out by available data. Importantly, this approach does not include a drug specific component to be included in the causality assessment.

A structured expert opinion process, such as that described by the Drug Induced Liver Injury Network (DILIN), has been shown to be superior to RUCAM [13]. We have previously shown that different drugs have different clinical DILI characteristics or phenotypes [14] and therefore, we speculate that one of the reasons that expert opinion is superior to RUCAM is that experts recognize drugs’ specific clinical phenotypes (i.e., its “signature” or typical characteristics). Unfortunately, a major limitation of the expert opinion approach is that it is not widely available in clinical practice and is thus not generalizable. Of note, broad phenotype characteristics for DILI events are provided in “LiverTox®” https://www.ncbi.nlm.nih.gov/books/NBK547852, however, there is no data-driven DILI phenotype currently in use for numeric assessment (scoring) of potential DILI cases.

Here, we hypothesized that certain clinical DILI features are typical for certain drugs, and that these make up a typical phenotype or signature, and such drug-specific DILI phenotypes could be used to develop a novel and quantitative DILI causality assessment tool (DILI-CAT) by incorporating data-driven drug-specific DILI phenotypes into the adjudication process. Therefore, we aimed to create a quantitative data-driven algorithm (DILI-CAT) based on drug-specific DILI phenotypes using characteristic DILI features. Based on our experience in DILI adjudication, we believe that clinical features including latency and biochemical patterns, are the most frequently used variables that inform the specific phenotype or characteristic features of a specific drug. Of biochemical features, the R-value (the ratio of alanine aminotransferase ALT to the upper limit of normal for (ALT) / alkaline phosphatase (ALP) to the upper limit of normal for ALP), is the biochemical variable most frequently utilized. Based on our own experience, we further hypothesized that the AST/ALT ratio may also be helpful in distinguishing a drug’s specific phenotype [15].

## Methods

We performed a literature search using PubMed to identify published case-series studies that included more than 10 cases prior to 2019 that reported clinical features in patients with DILI caused by a single specific drug. Case studies and series of various drugs have been reported in the literature, but very few have reported patient level data. We were only able to identify four case series that fulfilled the requirement of having detailed patient-level data for latency, defined as time between drug start and liver injury onset, R-value, and AST/ALT ratio at onset. The four studies identified included one study for each of the following four drugs: Cyproterone (n = 22, [16], amoxicillin-clavulanate (n = 35, [17], cefazolin (n = 19, [18], and *Polygonum multiflorum* (n = 18, [19].

### Design

We hypothesized that the closer a drug’s clinical characteristics to those same features that are published, the more likely the case is a bona fide DILI case due to a specific drug. In other words, the closer a case’s values are to the interquartile range (IQR) of values in published DILI cases for that drug, the more likely that injury is related to the drug in question.

We developed a quantitative “drug-specific” scoring system that allocates points based on the distribution of latencies, R-value at onset, and AST/ALT ratio at onset (in previously published case series [16–19]. Given our experience and the available data suggesting that age and gender are not important in informing a drug’s specific phenotype, we did not include these variables in the model. Separate DILI-CAT scoring was developed for each drug.

**Fig 1:**
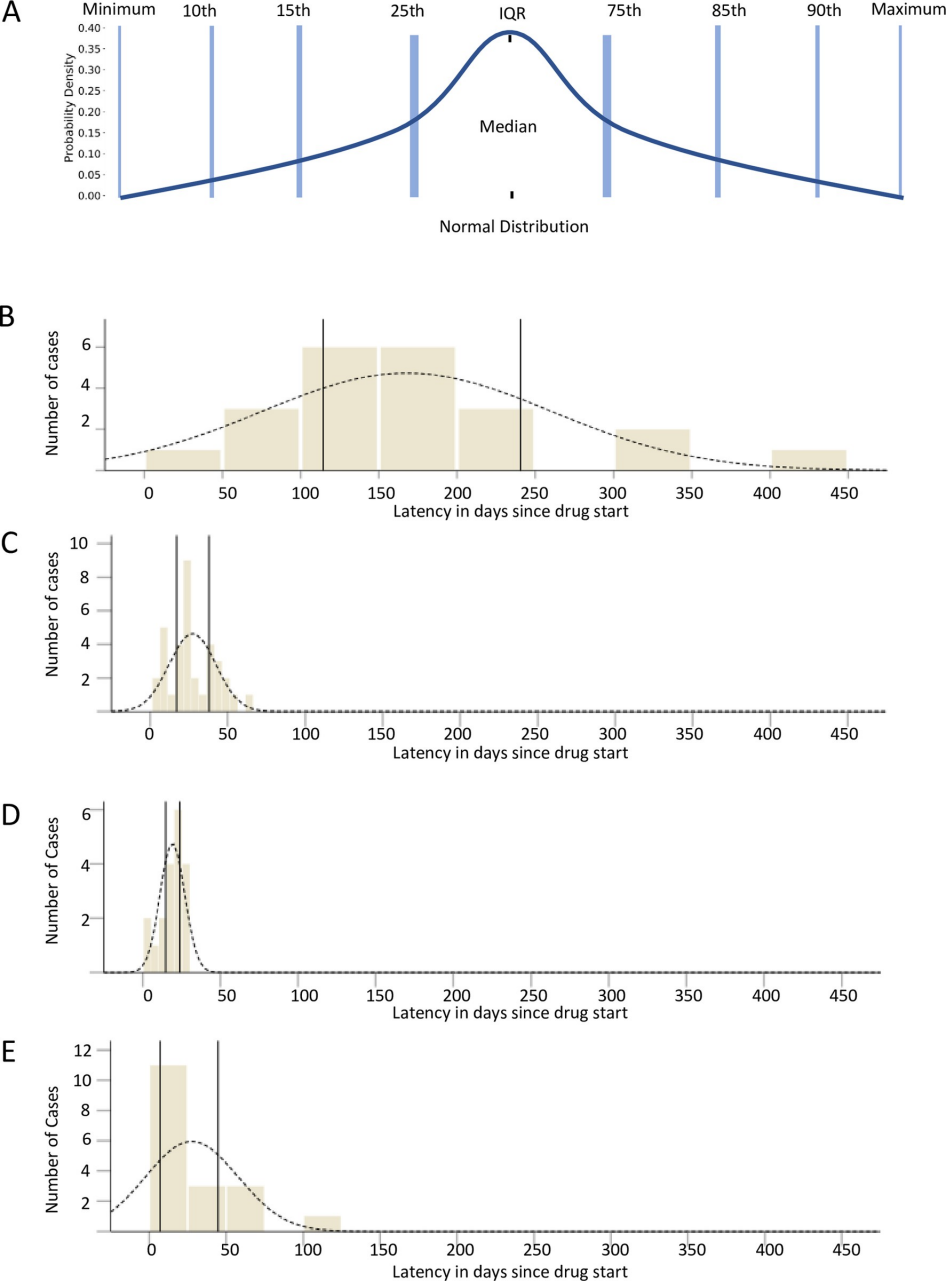
Frequency distribution of latency of cases compared to a normal distribution. Normal distribution (A) is shown compared to distribution of non-normal distribution of latency among DILI cases due to cyproterone (B), AMX/CLA (C), cefazolin (D), and *Polygonum multiflorum* (E). In panels B to E, frequency of cases is given on the Y axis and latency in days from drug start in the X axis; the vertical lines in panels B-E represent the interquartile range or 25^th^ and 75^th^ percentile. Abbreviations: AMX/CLA, amoxicillin/clavulanate; DILI, drug-induced liver injury.

In the model, points were allocated based on the closeness of the variable of interest for each specific drug to the IQR (also the “50% core interval”) (Fig 1 and S1a and S1b Fig in S1 Fig) as derived from known cases (patient/case level data) [16–19]. Proportionally fewer points were allocated when values for the variable of interest fell outside the IQR. A data value falling within the core interval was allocated 20 points (Tables 1 and S1). Values falling outside the IQR were given fewer points (Table 1). Deductions were given for values outside of the range of the values for respective drug’s phenotype range (Fig 1 and Tables 1 and S1). Additional deductions were also given when values were far outside the IQR; these were defined as “outliers” (see S1 Appendix).

**Table 1:** Relative point allocation according to value relative to the distribution of values within the respective case series.

Value within	Percentage of points to be allocated	Point allocation
IQR (25^th^ to 75^th^ percentile)	100%	20
25^th^ to 15^th^ percentile	50%	10
75^th^ to 85^th^ percentile		
15^th^ to 10^th^ percentile	25%	5
85^th^ to 90^th^ percentile		
10^th^ percentile to minimum of range	0%	0
90^th^ percentile to maximum of range		
Below minimum of range	-25%	-5
Above maximum of range		
Outlier*	-25%	-5
Both outlier and outside of range	-50%	-10

* defined as values far outside the range; a detailed definition of outlier is given in S1 Appendix.

Abbreviations: IQR, interquartile range.

The strategy that was ultimately utilized to generate a scoring system required several assumptions. First, we postulated that each of the four drugs chosen (or any other drug, for that matter) would exhibit differences in one or more of the three clinical features we believed to be most important in evaluation of DILI.

Furthermore, a weighting system was used in which the greatest differences in the 3 clinical features among different drugs were considered; this led to scoring that included specific “weighting”—latency-weighting, R-value‒weighting, or AST/ALT ratio‒weighting. Specifically, the clinical feature (latency, R-value at onset, or AST/ALT ratio) with the greatest difference among two drugs being compared received two-fold greater weight compared to the remaining two categories. The variation in each clinical feature for each comparison was assessed using a non-parametric Mann-Whitney rank test. Therefore, for the specific clinical feature with the greatest discriminating potential (defined by the lowest U-value in the Mann-Whitney rank test), that clinical feature would be weighted such that the DILI-CAT subscore value for the specific category would be doubled. For example, if for a specific drug, latency exhibited the greatest statistical difference compared to R-value or AST/ALT ratio, then latency points were doubled (i.e. weighted) so as to allow this clinical feature to become more important in assigning the DILI-CAT score. The terminology used for this preferential scoring was thus termed “latency weighting”, “R-value weighting” or “AST/ALT ratio weighting”.

### Statistics

Each drug’s phenotype was informed by the IQR, percentiles, maximum and minimum values, and definition of outlier values for each of the three clinical variables (latency, R-value, AST/ALT ratio).

A Mann-Whitney rank test was used to compare drug phenotypes for each of the three clinical variables to each other. Differences in variables were defined statistically. In brief, the smaller the Mann-Whitney “U-value”, the greater the difference, and a Mann-Whitney U value of “0” reflects complete separation of parameters between groups (i.e., the Mann-Whitney number comparing latency for cyproterone and cefazolin was zero, reflecting that all latencies for cyproterone were longer than any cefazolin latencies).

For each drug, a drug-specific DILI-CAT scoring was developed using the outlined scoring algorithm (Table 1) and the data derived from that respective drug. To compare drug-specific DILI-CAT performance, each drug was evaluated using its respective DILI-CAT scoring against the three other drugs, where the significance of difference was assessed using the Mantel-Haenszel test for trend considering five-point incremental scores as ordinal categories; in this test the higher the numerical value, the greater the difference. Furthermore, p-values below 0.05 were considered significant. Data handling was done using Microsoft^®^ Excel^®^, and IBM^®^ SPSS^®^ version 25 was used for statistical analysis.

## Results

### Drug induced liver injury phenotypes

The DILI phenotypes were defined by the following: (1) latency (in days), (2) R-value, and (3) AST/ALT ratio for each of the 4 drugs included in this analysis differed (Table 2A–2D).

**Table 2:** Drug phenotypes described by latency, R-value & AST/ALT ratio with interquartile range and percentiles.

A	Cyproterone (n = 22)		
	Latency*	R-Value	AST/ALT ratio
Median	150	12.4	0.8
IQR	114–240	8.8–18	0.7–1.2
Range	33–425	1–30	0.2–2.1
B	AMX-CLA (n = 35)		
	Latency*	R-Value	AST/ALT ratio
Median	25.5	1.44	0.7
IQR	17–38	0.6–2.9	0.4–0.9
Range	4–63	0.2–14	0.2–1.9
C	Cefazolin (n = 19)		
	Latency*	R-Value	AST/ALT ratio
Median	20	1.57	0.4
IQR	18–26	1.1–3.4	0.4–0.7
Range	6–29	0.5–11	0.2–1.2
D	*Polygonum multiflorum* (n = 18)		
	Latency*	R-Value	AST/ALT ratio
Median	20	10.9	0.5
IQR	7–45	6.8–14.3	0.4–0.3
Range	1–120	2.8–26	0.3–2.5

*Latency is in days from drug start to DILI onset. ALT: alanine transaminase; AMX/CLA: amoxicillin/clavulanate; AST: aspartate transaminase; R-value: ALT in ULN (upper limits of normal) divided by alkaline phosphatase in ULN.

### Quantitative differences among drug phenotypes

The latency for cyproterone was significantly longer (median 150 days, p<0.001 Table 3A) than that for the other three drugs (which ranged from a median of 26 days for AMX/CLA, median 20 days for cefazolin and *Polygonum multiflorum* (Table 2 and Fig 1).

**Table 3:** Differences in phenotypes (Latency, R-value & AST/ALT ratio) among different drugs. **A.** Mann-Whitney U-value and p-value for comparison of cyproterone versus AMX/CLA, cefazolin and *Polygonum multiflorum*. **B.** Mann-Whitney U-value and p-value for comparison of AMX/CLA versus cyproterone, cefazolin, and *Polygonum multiflorum*. **C.** Mann-Whitney U-value and p-value for comparison of cefazolin versus cyproterone, AMX/CLA, and *Polygonum multiflorum*. **D.** Mann-Whitney U-value and p-value for comparison of *Polygonum multiflorum* versus cyproterone, cefazolin, and AMX/CLA.

**A**	**Cyproterone (n = 22)**		
	Latency*	R-Value	AST/ALT ratio
**AMX/CLA**			
Mann-Whitney U-value	**12.5**	65	289
p-value	**<0.001**	<0.000	0.116
**Cefazolin**			
Mann-Whitney U-value	**0**	34	100
p-value	**<0.001**	<0.000	0.004
**Polygonum multiflorum**			
Mann-Whitney U-value	**12**	164	101
p-value	**<0.001**	0.355	0.008
**B**	**AMX/CLA (n = 35)**		
	Latency*	R-Value	AST/ALT ratio
**Cyproterone**			
Mann-Whitney U value	**12.5**	65.0	289.0
p-value	**<0.001**	0.000	0.116
**Cefazolin**			
Mann-Whitney U-value	241	276	**218**
p-value	0.097	0.306	**0.038**
**Polygonum multiflorum**			
Mann-Whitney U-value	262	**41**	232
p-value	0.319	**<0.001**	0.119
**C**	**Cefazolin (n = 19)**		
	Latency*	R-Value	AST/ALT ratio
**Cyproterone**			
Mann-Whitney U	**0**	34	100
p-value	**<0.001**	0.000	0.004
**AMX/CLA**			
Mann-Whitney U	241	276	**218**
p-value	0.097	0.306	**0.038**
**Polygonum multiflorum**			
Mann-Whitney U	164	**17**	146
p-value	0.842	**<0.001**	0.447
**D**	***Polygonum multiflorum* (n = 18)**		
	Latency*	R-Value	AST/ALT ratio
**Cyproterone**			
Mann-Whitney U	**12**	164	101
p-value	**<0.001**	0.355	0.008
**AMX/CLA**			
Mann-Whitney U	262.000	**41**	232
p-value	0.319	**<0.001**	0.119
**Cefazolin**			
Mann-Whitney U	171	**17**	146
p-value	0.843	**<0.001**	0.447

*Latency in days from drug start to DILI onset; ALT: alanine transaminase; AMX/CLA, amoxicillin/clavulanate; AST: aspartate transaminase; DILI, drug-induced liver injury; R-value: ALT in ULN (upper limits of normal) divided by alkaline phosphatase in ULN. Bold numbers in shaded areas represent the most significant differences.

The R-values also were similar for cyproterone and *Polygonum multiflorum*, (median 12.4 and 10.9, Table 2A and 2D; p = 0.355 Table 3A and 3D), on one side and for AMX-CLA and cefazolin on the other side (median 1.4 and 1.6 Table 2B and 2C, p = 0.31, Table 3B and 3C), (S1a Fig in S1 Fig). However, R-value differed significantly comparing cyproterone or *Polygonum multiflorum* to AMX-CLA or cefazolin, respectively (Table 3A–3D).

The AST/ALT ratio was significantly different only between AMX/CLA and cefazolin (median 0.67 versus 0.42 Table 2B and 2C, p = 0.038, Table 3B and 3C and S1b Fig in S1 Fig).

### Use of DILI-CAT to assess drug specific phenotypic differences

The DILI-CAT utilizes weighting of individual phenotypic features (latency, R-value, and AST/ALT ratio, as described in the Methods). That is to say that in order to most accurately differentiate signatures among drugs, when a clear distinction in either latency, R-value, or AST/ALT ratio was identified, then this specific clinical feature was weighted to a greater degree than the other categories, so as to allow better differentiation among the drugs.

Cyproterone showed the greatest difference in latency compared to the other three drugs (p<0.001, Table 3). *Polygonum multiflorum* differed from cyproterone most strongly in terms of latency (p<0.001, Table 3A and 3D) and differed from AMX-CLA and cefazolin significantly only in R-value (p<0.001, Table 3B–3D). AMX-CLA and cefazolin differed significantly only in the AST/ALT ratio from each other (p = 0.038, Table 3B and 3C). Based on the respective greatest difference, as defined by lowest U-value (Table 3A–3D), the following weighting was applied:

- For cyproterone, a latency-weighted (thus latency valued double) DILI-CAT was applied for comparison against all other three drugs.
- For both AMX-CLA and cefazolin, a latency-weighted DILI-CAT was applied against cyproterone, an R-value‒weighted DILI-CAT against *Polygonum multiflorum*, and, finally, AMX/CLA and cefazolin were compared to each other using an AST/ALT ratio‒weighted AMX/CLA-DILI-CAT or cefazolin-DILI-CAT, respectively.
- For *Polygonum multiflorum*, a latency-weighted *Polygonum multiflorum*-DILI-CAT was applied for comparison against cyproterone, but an R-value‒weighted *Polygonum multiflorum*-DILI-CAT was applied for comparison against both AMX-CLA and cefazolin.

### Cyproterone DILI-CAT

In order to create a cyproterone-DILI-CAT scoring, as outlined in the Methods, points were allocated based on latency, R-value, and AST/ALT ratio. As outline in the section “phenotype differences”, for cyproterone, the U value was lowest and thus displaying the greatest difference for latency when comparing cyproterone to all other drugs (Table 3A).

The difference in cyproterone-DILI-CAT scores for respective clinical feature and weighted cyproterone-DILI-CAT score for the 4 different drugs were evaluated using the Mantel-Haenszel test for trend (Table 4; for case level data see S2a Table in S2 Table and S2a Fig in S2 Fig). As can be seen, median DILI-CAT points allocated for each of the 3 clinical DILI features varied from -7.5 to 10 for the drugs other than cyproterone. (left side of Table 4), and from 0 to 32.5 for weighted cyproterone-DILI-CAT scores (right side of Table 4). Because latency was the strongest differentiating clinical feature defined by lowest U-value (Table 3A), the (median) latency weighted DILI-CAT score (median of 47.5) becomes the final median cyproterone-DILI-CAT score for cyproterone and was significantly different from all other drugs evaluated here (Table 4). These data also emphasize that *Polygonum multiflorum* had the closest clinical phenotype to cyproterone.

**Table 4:** Point scoring for cyproterone. (Median scores for cyproterone vs. other drugs using cyproterone-DILI-CAT).

*	Median cyproterone-DILI-CAT Subscores for Each Clinical Feature			Median cyproterone-DILI-CAT Weighted Scores		
	Latency	R-Value	AST/ALT ratio	Latency weighted	R-Value weighted	AST/ALT ratio weighted
**Cyproterone (n = 22)**	**20**	**20**	**20**	**47.5**	**55**	**50**
AMX/CLA (n = 35)	-5	-5	10	5	0	20
Cefazolin (n = 19)	-5	-5	10	5	5	20
PM (n = 18)	-7.5	10	10	15	32.5	30
* numbers shown are median points allocated for each drug						
	p-values based on Mantel-Haenszel test for trend for Cyproterone versus the other three drugs					
AMX/CLA (n = 35)	**<0.001**	**<0.001**	0.785	**<0.001**	**<0.001**	**<0.001**
Cefazolin (n = 19)	**<0.001**	**<0.001**	**0.595**	**<0.001**	**<0.001**	**<0.001**
PM (n = 18)	**<0.001**	0.984	0.352	**<0.001**	**0.004**	**0.001**

Abbreviations: AMX/CLA, amoxicillin/clavulanate; DILI-CAT: drug-induced liver injury causality assessment tool; PM: *Polygonum multiflorum*.

### AMX/CLA DILI-CAT

AMX/CLA is known to have a wide variation in clinical phenotype and can cause a wide array of biochemical abnormalities. Therefore, as expected, it had a more complex clinical pattern and generally differed modestly from the other drugs. The most significant differences between AMX/CLA and the other drugs depended on the specific comparator drug. AMX/CLA differed from cyproterone strongest in latency (see U values in Table 3B) and therefore a latency weighting was used to differentiate AMX/CLA from cyproterone (with latency weighted AMX/CLA-DILI-CAT scores resulting in 55 points for AMX/CLA and -7.5 for cyproterone (p<0.001, Table 5 and S2b Table in S2 Table for case level data; S2b Fig in S2 Fig).

AMX-CLA differed from *Polygonum multiflorum* significantly only in R-value (U-value of 41, p<0.001, Table 3B) and therefore an R-value weighting AMX/CLA-DILI-CAT would yield the strongest differentiation from other drugs resulting in 60 points for AMX/CLA and -2.5 for *Polygonum multiflorum* (p<0.001, Table 5 and see S2b Table in S2 Table for case level data, S2b Fig in S2 Fig).

**Table 5:** Point scoring for AMX-CLA. (Median scores for AMX-CLA vs. other drugs using AMX/CLA-DILI-CAT).

	Median AMX/CLA-DILI-CAT Subscore for each Clinical Feature			Median AMX/CLA-DILI-CAT weighted Scores		
*	Latency	R-Value	AST/ALT ratio	Latency weighted	R-Value weighted	AST/ALT ratio weighted
**AMX/CLA (n = 35)**	**20**	**20**	**20**	**55**	**60**	**60**
Cyproterone (n = 22)	-10	-5	20	-7.5	-2.5	20
Cefazolin (n = 19)	20	20	10	70	60	60
PM (n = 18)	2.5	-5	15	25	10	27.5
* numbers shown are median points allocated for each drug						
	Mantel-Haenszel test for trend p-values for AMX/CLA versus the other three drugs					
Cyproterone (n = 22)	**<0.001**	**<0.001**	0.785	**<0.001**	<0.001	<0.001
Cefazolin (n = 19)	0.195	0.379	0.356	0.292	0.416	0.866
PM (n = 18)	**0.003**	**<0.001**	0.989	<0.001	**<0.001**	<0.001

Abbreviations: AMX/CLA, amoxicillin/clavulanate; DILI-CAT: drug-induced liver injury causality assessment tool; PM: *Polygonum multiflorum*.

AMX/CLA and cefazolin were relatively similar in terms of their latency and R-value (U-value of 227 for latency and 276 for R-value, Table 3B), but differed from each other in AST/ALT ratio (U-value of 218, p = 0.038,). Therefore, an AST/ALT ratio weighting AMX/CLA-DILI-CAT was used resulting in 60 points for both AMX/CLA and cefazolin (Table 5 and see S2a Table in S2 Table for case level data, S2b Fig in S2 Fig) and no difference was seen between AMX/CLA and cefazolin using AMX/CLA-DILI-CAT.

### Cefazolin DILI-CAT

Using the cefazolin derived cefazolin-DILI-CAT, cefazolin was similar to AMX/CLA in all three clinical categories with the smallest U-value being found for AST/ALT ratio (U-value 218, p = 0.038, Table 3C). Therefore, an AST/ALT ratio weighted cefazolin-DILI-CAT was to be used for Cefazolin vs. AMX/CLA. While the AMX/CLA-derived AMX/CLA-DILI CAT score did not separate AMX/CLA from cefazolin (p>0.4, Table 5), a cefazolin-derived AST/ALT ratio weighted cefazolin-DILI-CAT was able to separate cefazolin from AMX/CLA with a median 60 DILI-CAT score for cefazolin vs. 40 for AMX/CLA (p = 0.008; Table 6 and S2c Table in S2 Table case level details; S2c Fig in S2 Fig). This is because the cefazolin phenotype shows less variation in latency, R-value and AST/ALT ratio compared to the AMX/CLA phenotype, where more AMX/CLA cases overlap with cefazolin’s phenotype but not vice versa (Table 2 and Fig 1 and S1a, S1b Fig in S1 Fig).

**Table 6:** Point scoring for cefazolin. (Median scores for Cefazolin vs. other drugs using cefazolin-DILI-CAT).

	Median cefazolin-DILI-CAT Subscores for each Clinical Feature			Median cefazolin-DILI-CAT weighted Scores		
*	Latency	R-Value	AST/ALT ratio	Latency weighted	R-Value weighted	AST/ALT ratio weighted
**Cefazolin (n = 19)**	**20**	**20**	**20**	**60**	**60**	60
Cyproterone (n = 22)	-10	-10	10	-12.5	-10	0
AMX-CLA (n = 35)	0	10	20	30	40	45
PM (n = 18)	0	-7.5	20	7.5	7.5	**25**
* numbers shown are median points allocated for each drug						
P-values for Cefazolin versus	Mantel-Haenszel test for trend					
Cyproterone (n = 22)	**<0.001**	**<0.001**	**0.045**	**<0.001**	**<0.001**	**<0.001**
AMX-CLA (n = 35)	**0.011**	0.082	0.244	**0.004**	**0.011**	**0.011**
PM (n = 18)	**<0.001**	**<0.001**	0.990	**<0.001**	**<0.001**	**<0.001**

Abbreviations: AMX/CLA, amoxicillin/clavulanate; DILI-CAT: drug-induced liver injury causality assessment tool; PM: *Polygonum multiflorum*.

Cefazolin differed from cyproterone, most strongly in latency (U-value 0 indicating no overlap in latency between the two drug, p<0.001, Table 3C) with a significantly different median latency weighted cefazolin-DILI-CAT score of 60 for cefazolin vs. -12.5 for cyproterone (p<0.001, Table 6 and see S2c Table in S2 Table for case level data; S2c Fig in S2 Fig).

Cefazolin differed significantly from *Polygonum multiflorum* only in R-value (U-value for R-value 17, p<0.001, Table 3C) Applying the R-value weighting cefazolin-DILI-CAT score, cefazolin differed significantly from *Polygonum multiflorum* with a median 60 points for cefazolin vs. a median score of 7.5 for *Polygonum multiflorum* (Table 6 and see S2c Table in for case level data; S2c Fig)).

### *Polygonum multiflorum* DILI-CAT

*Polygonum multiflorum* was most different from cyproterone in the latency category (U-value 12, p<0.001, Table 3D). Therefore, the *Polygonum multiflorum* derived latency weighted *Polygonum multiflorum*-DILI-CAT score is to be used resulting in median 57.5 points for *Polygonum multiflorum* compared to 7.5 points for cyproterone (Table 7 and S2d Table in S2 Table and S2d Fig in S2 Fig).

**Table 7:** Point scoring for *Polygonum Multiflorum* (PM). (Median scores for *Polygonum Multiflorum* (PM) vs. other drugs using *Polygonum multiflorum*-DILI-CAT).

	Median Score for each Clinical Feature			Median *Polygonum multiflorum* DILI-CAT weighted DILI-CAT Scores		
*	Latency	R-Value	AST/ALT ratio	Latency weighted	R-value weighted	AST/ALT ratio weighted
**PM (n = 18)**	**20**	**20**	**20**	**57.5**	**60**	**57.5**
Cyproterone (n = 22)	-10	15	5	7.5	32.5	25
AMX-CLA (n = 35)	20	-5	10	45	20	40
Cefazolin (n = 19)	20	-5	10	45	20	35
* numbers shown are median points allocated for each drug						
	Mantel-Haenszel test for trend P-values for *Polygonum multiflorum* (n = 18) versus the other three drugs					
Cyproterone (n = 22)	**<0.001**	0.282	**0.033**	**<0.001**	**0.001**	**<0.001**
AMX-CLA (n = 35)	0.031	<0.001	0.169	0.011	**<0.001**	0.002
Cefazolin (n = 19)	0.013	<0.001	0.07	0.036	**<0.001**	0.002

Abbreviations: AMX/CLA, amoxicillin/clavulanate; DILI-CAT: drug-induced liver injury causality assessment tool; PM: *Polygonum multiflorum*.

In contrast, *Polygonum multiflorum* was most significantly distinct from AMX/CLA and cefazolin in R-value (U-value of 41 and 17, respectively, p<0.001, Table 3). Therefore, a *Polygonum multiflorum* derived R-value‒weighted *Polygonum multiflorum*-DILI-CAT was to be used and demonstrated median 60 points for *Polygonum multiflorum* vs. median 20 points for each AMX/CLA as well as cefazolin (p<0.001, Table 7 and S2d Fig in S2 Fig).

## Discussion

Here, we have demonstrated a data-driven approach to develop a DILI causality tool (DILI-CAT) that can be used to create a quantitative drug-specific DILI phenotype. We demonstrate that drugs differ significantly in their phenotypes and that our algorithmic approach allows for differentiation of DILI caused by different drugs. Therefore, this tool has the potential to enhance DILI causality assessment.

RUCAM, the commonly used causality assessment method (tool) developed almost three decades ago [10], is often considered the most reliable approach to DILI causality assessment when an expert opinion assessment is not available [13]. However, neither RUCAM nor any of the other currently available causality assessment tools uses a drug-specific approach. An expert opinion approach is considered superior to RUCAM, which is likely because experts probably consider a drug’s phenotype. Implicit in the findings presented here is that allowing a formal process for inclusion of a drug phenotype enhances the DILI adjudication process by including phenotypic characteristics of drug-specific DILI. We speculate that although this should not necessarily replace RUCAM or expert opinion as causality tools, this approach should be extremely helpful to experts and, perhaps to an even greater degree, to nonexperts [20].

An algorithmic data-driven and drug-specific diagnostic tool such as DILI-CAT has several attractive features. First and most importantly, DILI-CAT is data-driven, using available data on a drug’s known DILI characteristics. Further, it can be optimized via weighting of specific variables, which will allow for better discrimination between different drugs. Additionally, other features that are part of a drug’s phenotype might be added in the mathematical algorithm. For example, the intrinsic propensity for hepatotoxicity of a drug (i.e., the likelihood or probability that a specific drug would cause liver injury) could be included (S2–S4 Appendices and S2 Table) based on published literature [21–23] or perhaps a generally available source such as LiverTox® (https://www.ncbi.nlm.nih.gov/books/NBK547852). As an alternative to ranking hepatotoxicity by number of published cases might be to rank based on a drug’s intrinsic propensity for hepatotoxicity, including for example being given in a high daily dose or perhaps its lipophilicity [24].

Scoring for competing causes in DILI-CATs could also be included, allowing for grading of individual drugs along a causality scale (S2 Appendix). Further, the flexible format of DILI-CAT allows it to be programmed for use by any drug, as long as the DILI phenotype of a drug can be characterized (e.g., with a sufficient number of known DILI cases to estimate percentiles of the drug-specific features). Finally, the approach should be considered a “living” process, meaning that additional cases could be added as more published cases become available so as to create a more robust DILI signature.

We recognize limitations of the current version of DILI-CAT as well as opportunities to enhance it. For one, we chose to examine latency, R-value, or AST/ALT ratio as important clinical elements of the drug signature. While this was based on sound rationale, and we chose to limit signature assessment to these 3 simple features to emphasize the simplicity of the approach, there is no reason other elements of a signature could not be included (i.e. such as genetics/HLA genotypes and gender, the latter is generally not considered in adjudication and is likely relevant for only a few drugs), as well as more traditional clinical parameters such as dechallenge, competing drugs, inherent hepatotoxicity of the drug, etc…). Compared to a general tool for adjudication where even a first case of liver injury can be assessed, DILI-CAT approach depends on previously identified cases, though the number required will need to be evaluated with future series as more patient level data will become available. An important limitation of DILI-CAT is that some drugs have overlapping phenotypes, such as was the case with cefazolin and AMX/CLA; in this situation, DILI-CAT will be unable to provide a clear distinction between drugs in question with similar quantitative phenotypes.

Another limitation is that DILI-CAT depends on having available cases with which to develop specific drug signatures. In an analysis of 671 distinct drugs or entities, 20% of drugs reported to have caused DILI had at least 12 reported cases [22, 23], suggesting that phenotypes can be developed for at least this proportion of drugs (it should be emphasized that in terms of overall case numbers, these 20% of drugs make up a large proportion of the total number of DILI events). An additional consideration is that the number of cases needed to develop a robust signature will depend on the consistency of the drug’s phenotype. The more variable the phenotype, the more cases that are likely to be required to generate a precise picture of a drug’s signature. We used case series, but a phenotype can also be retrieved from combining various studies or case reports or case series if the required information is available. The more cases that are available for inclusion into model development, the more accurate a described phenotype is likely to became. This approach can be applied to a small number of cases to start with, so long as the cases are well-characterized, providing sufficient information for the phenotyping [23]. Another limitation is the lack of consideration of host factors and drug-host interactions; host factors, including age, sex, genetic variants, comorbidities, and concomitant medications may modify DILI phenotypes, via modifying cellular stress response, immune response, and tissue repair. This limitation cannot be solved presently due to the limited knowledge of drug-host interactions and drug-drug interactions in DILI phenotypes.

In the future, we envision a staggered approach to DILI causality assessment. First, the likelihood of DILI could be assessed using an algorithmic methodology such as that presented here, and secondly laboratory testing could be used for confirmation. While lymphocyte transformation test (LTT) is recommended in the Japanese DDW-J scoring for DILI [12], it is unclear whether this assay is reproducible enough to be used [25]. A novel promising approach is based on assay of blood derived monocytes that are transformed into hepatocyte like cells [26]. In several studies, this test has shown promise as a confirmatory assay [27–29].

In summary, we have presented an objective and data-driven drug-specific tool (DILI-CAT) that represents a novel and substantial step forward in DILI causality assessment. This approach is likely to be extremely useful for clinicians who are not experts in DILI causality assessment, and it also has the potential to improve expert adjudication of DILI.

## Supporting information

S1 Fig This figures shows the distribution for R-value (a) and AST/ALT ratio (b) for all four drugs respectively.
(DOCX)

S2 Fig S2a Fig. This figure shows the distribution of Cyproterone-DILI-CAT scores compared to A) the AMX/CLA (Amoxicillin.clavulunaic) scores, b) cephazolin scores and c) Polygonum multiflorum scores); S2b Fig. This figure shows the distribution of AMX/CLA (Amoxicillin.clavulunaic)-DILI-CAT scores compared to A) the Cyproterone scores, b) cephazolin scores and c) Polygonum multiflorum scores); S2c Fig. This figure shows the distribution of cephazolin-DILI-CAT scores compared to A) the Cyproterone scores, b) AMX/CLA (Amoxicillin.clavulunaic)-scores and c) Polygonum multiflorum scores); S2d Fig. This figure shows the distribution of Polygonum multiflorum -DILI-CAT scores compared to A) the Cyproterone scores, b) AMX/CLA (Amoxicillin.clavulunaic)-scores and c) cephazolin scores).
(ZIP)

S1 Table This table shows the examples for point allocation for latency throughout the first 100 days. (DOCX)

S2 Table S2a Table. Cyproterone DILI-CAT: This table shows the Cyproterone derived DILI-CAT-scoring algorithm comparing the cyproterone cases to the cases of the other drugs using the Cyproterone derived DILI-CAT; S2b Table. AMX/CLA DILI-CAT: This table shows the Amoxicilling/Clavulunaic (AMX/CLA) derived DILI-CAT-scoring algorithm comparing the AMX/CLA cases to the cases of the other drugs using the AMX/CLA derived DILI-CAT; S2c Table. Cefazolin DILI-CAT: This table shows the cefazolin derived DILI-CAT-scoring algorithm comparing the cefazolin cases to the cases of the other drugs using the cefazolin derived DILI-CAT; S2d Table. Polygonum multiflorum DILI-CAT: This table shows the Polygonum multiflorum derived DILI-CAT-scoring algorithm comparing the Polygonum multiflorum cases to the cases of the other drugs using the Polygonum multiflorum derived DILI-CAT cefazolin.
(ZIP)

S1 Appendix This material shows the outlier definition used. (DOCX)

S2 Appendix This material indicates the point allocation for competing causes and the lack thereof (-25 to 25 points) to be used for a complete causality adjudication. (DOCX)

S3 Appendix This material indicates the point allocation for hepatotoxicity potential of a respective drug (0–20 points) to be used for a complete causality adjudication. (DOCX)

S4 Appendix This material indicates theoretical maximal positive and negative scores possible for a DILI-CAT scoring in its current iteration. (DOCX)
