# Supplementary material for: A novel quantitative computer-assisted drug-induced liver injury causality assessment tool (DILI-CAT)
Source: PLoS One. 2022 Sep 29;17(9):e0271304. doi: 10.1371/journal.pone.0271304 (PMC9521919; doi:10.1371/journal.pone.0271304)
Supplement: S1 Fig — This figures shows the distribution for R-value (a) and AST/ALT ratio (b) for all four drugs respectively. (DOCX) [file pone.0271304.s001.docx]

**Supplemental Figure 1a. Frequency distribution of R-value of cases for 4 different drugs for cyproterone (A), AMX/CLA (B), cefazolin (C), and polygonum multiflorum (D) Frequency of cases is given on the Y axis and R-value as numbers in the X axis; the vertical lines in figures A-D represent the interquartile range or 25^th^ and 75^th^ percentile.**

AMX/CLA, amoxicillin/clavulanate.

**
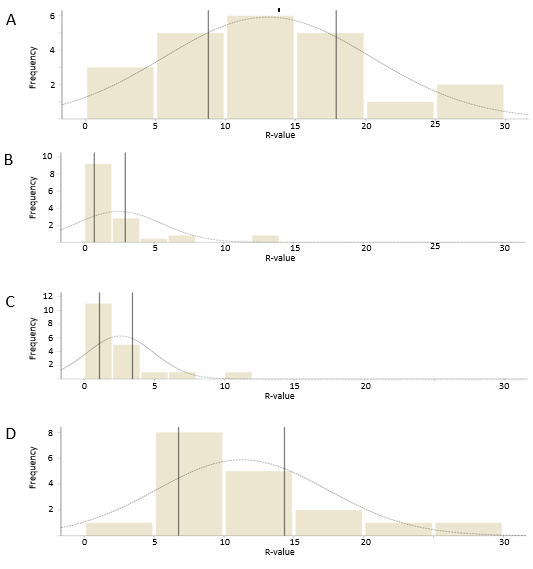
**

**Supplemental Figure 1b**. Frequency distribution of AST:ALT ratio of cases for 4 different drugs for cyproterone (A), AMX/CLA (B), cefazolin (C), and polygonum multiflorum (D). Frequency of cases is given on the Y axis and AST:ALT ratio as numbers in the X axis; the vertical lines in figures A-D represent the interquartile range or 25^th^ and 75^th^ percentile.

ALT, alanine aminotransferase; AMX/CLA, amoxicillin/clavulanate; AST, aspartate aminotransferase.

**
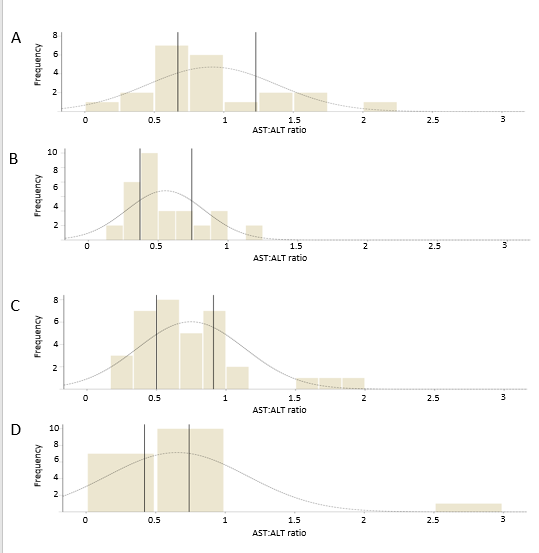
**
