# Supplementary material for: A novel quantitative computer-assisted drug-induced liver injury causality assessment tool (DILI-CAT)
Source: PLoS One. 2022 Sep 29;17(9):e0271304. doi: 10.1371/journal.pone.0271304 (PMC9521919; doi:10.1371/journal.pone.0271304)
Supplement: S2 Fig — S2a Fig. This figure shows the distribution of Cyproterone-DILI-CAT scores compared to A) the AMX/CLA (Amoxicillin.clavulunaic) scores, b) cephazolin scores and c) Polygonum multiflorum scores); S2b Fig. This figure shows the distribution of AMX/CLA (Amoxicillin.clavulunaic)-DILI-CAT scores compared to A) the Cyproterone scores, b) cephazolin scores and c) Polygonum multiflorum scores); S2c Fig. This figure shows the distribution of cephazolin-DILI-CAT scores compared to A) the Cyproterone scores, b) AMX/CLA (Amoxicillin.clavulunaic)-scores and c) Polygonum multiflorum scores); S2d Fig. This figure shows the distribution of Polygonum multiflorum -DILI-CAT scores compared to A) the Cyproterone scores, b) AMX/CLA (Amoxicillin.clavulunaic)-scores and c) cephazolin scores). (ZIP) [file pone.0271304.s002.zip › Sup Fig 2a.pptx]

## Slide 1
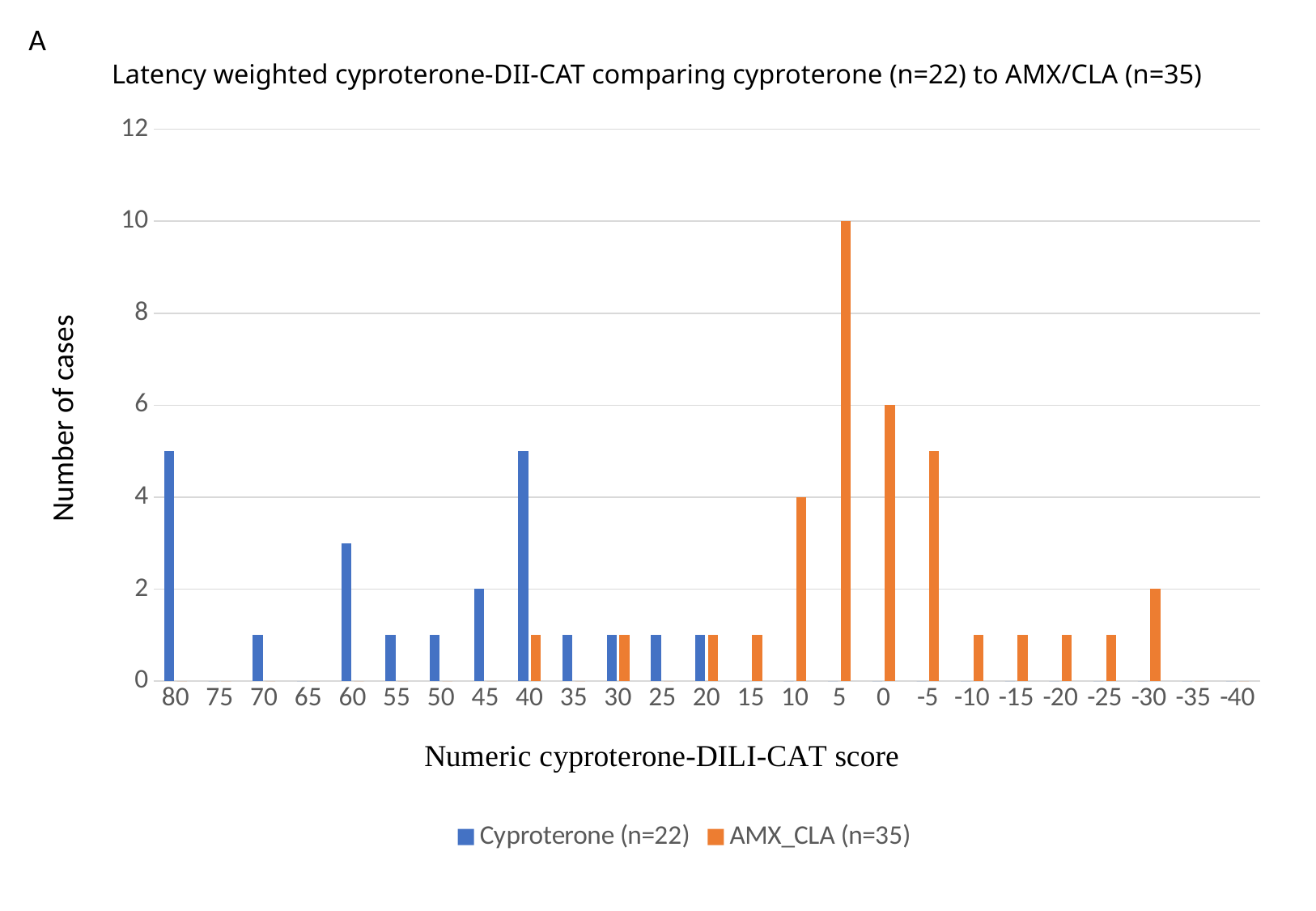

A
Latency weighted cyproterone-DII-CAT comparing cyproterone (n=22) to AMX/CLA (n=35)
### Chart
| Category | Cyproterone (n=22) | AMX_CLA (n=35) |
|---|---|---|
| 80 | 5.0 | 0.0 |
| 75 | 0.0 | 0.0 |
| 70 | 1.0 | 0.0 |
| 65 | 0.0 | 0.0 |
| 60 | 3.0 | 0.0 |
| 55 | 1.0 | 0.0 |
| 50 | 1.0 | 0.0 |
| 45 | 2.0 | 0.0 |
| 40 | 5.0 | 1.0 |
| 35 | 1.0 | 0.0 |
| 30 | 1.0 | 1.0 |
| 25 | 1.0 | 0.0 |
| 20 | 1.0 | 1.0 |
| 15 | 0.0 | 1.0 |
| 10 | 0.0 | 4.0 |
| 5 | 0.0 | 10.0 |
| 0 | 0.0 | 6.0 |
| -5 | 0.0 | 5.0 |
| -10 | 0.0 | 1.0 |
| -15 | 0.0 | 1.0 |
| -20 | 0.0 | 1.0 |
| -25 | 0.0 | 1.0 |
| -30 | 0.0 | 2.0 |
| -35 | 0.0 | 0.0 |
| -40 | 0.0 | 0.0 |Number of cases

## Slide 2
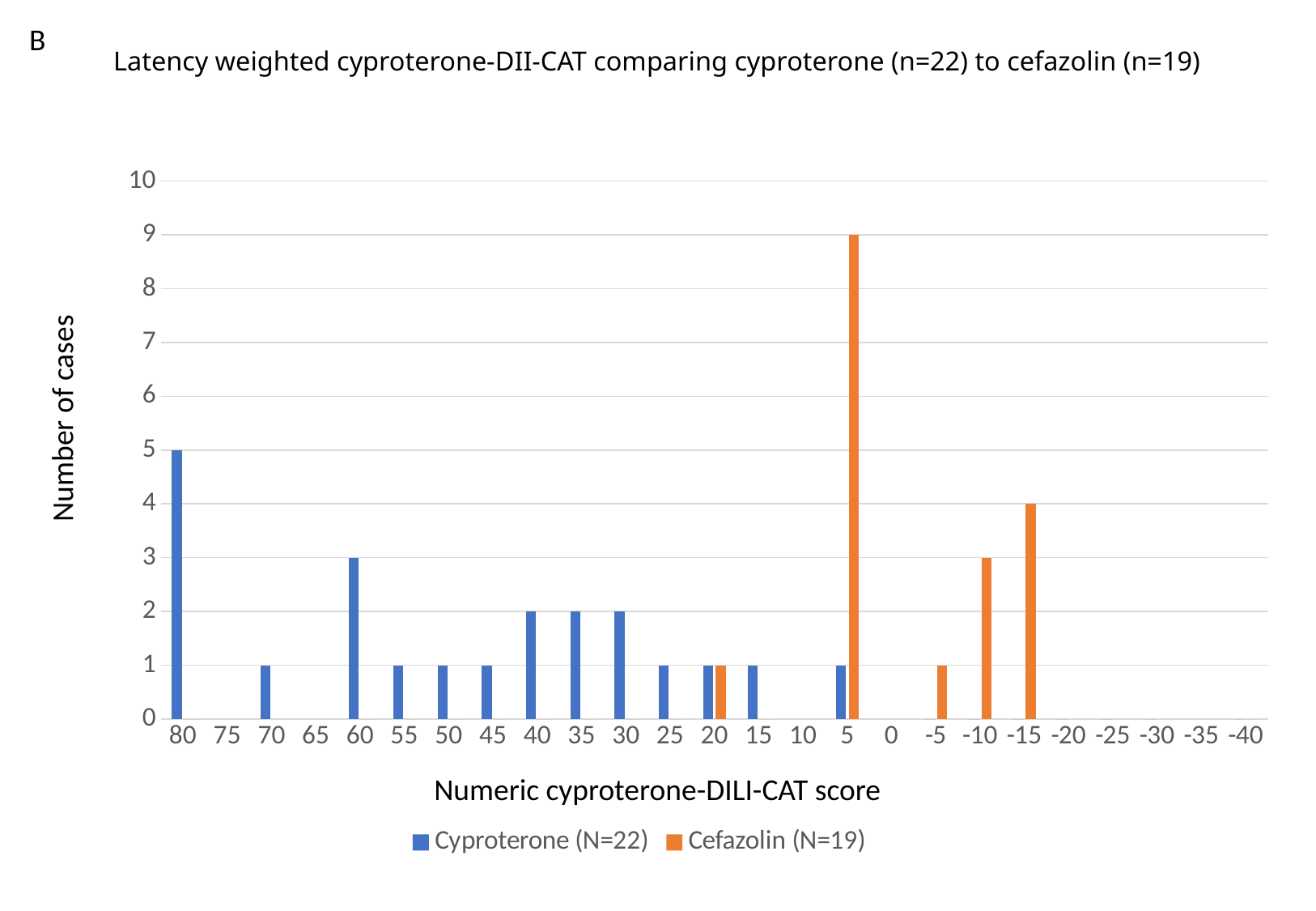

B
# Latency weighted cyproterone-DII-CAT comparing cyproterone (n=22) to cefazolin (n=19)
### Chart
| Category | Cyproterone (N=22) | Cefazolin (N=19) |
|---|---|---|
| 80 | 5.0 | 0.0 |
| 75 | None | None |
| 70 | 1.0 | None |
| 65 | None | None |
| 60 | 3.0 | 0.0 |
| 55 | 1.0 | 0.0 |
| 50 | 1.0 | 0.0 |
| 45 | 1.0 | 0.0 |
| 40 | 2.0 | 0.0 |
| 35 | 2.0 | 0.0 |
| 30 | 2.0 | 0.0 |
| 25 | 1.0 | 0.0 |
| 20 | 1.0 | 1.0 |
| 15 | 1.0 | 0.0 |
| 10 | None | None |
| 5 | 1.0 | 9.0 |
| 0 | None | None |
| -5 | 0.0 | 1.0 |
| -10 | 0.0 | 3.0 |
| -15 | 0.0 | 4.0 |
| -20 | 0.0 | None |
| -25 | 0.0 | None |
| -30 | 0.0 | None |
| -35 | None | None |
| -40 | 0.0 | 0.0 |Number of cases
Numeric cyproterone-DILI-CAT score

## Slide 3
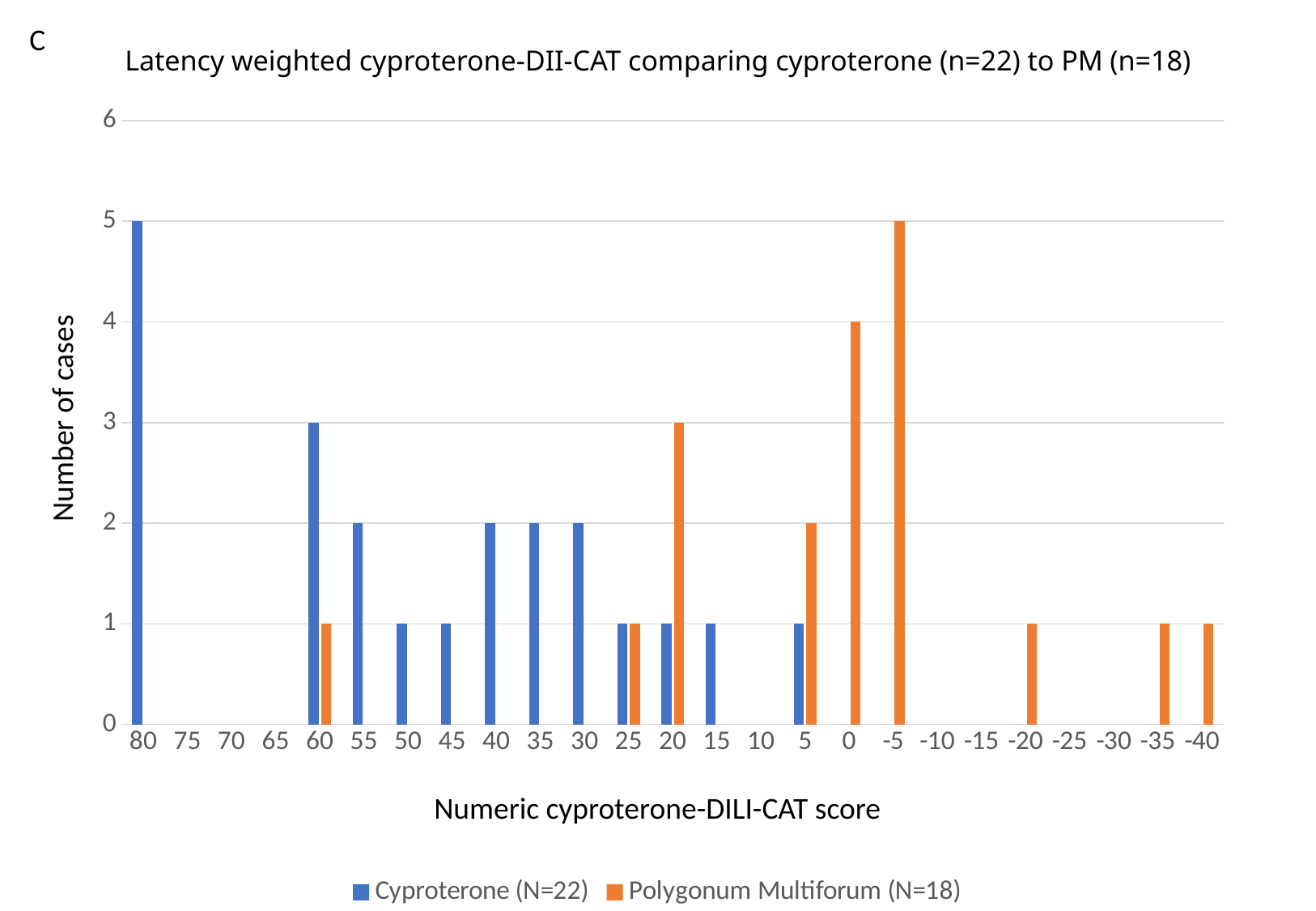

C
# Latency weighted cyproterone-DII-CAT comparing cyproterone (n=22) to PM (n=18)
### Chart
| Category | Cyproterone (N=22) | Polygonum Multiforum (N=18) |
|---|---|---|
| 80 | 5.0 | 0.0 |
| 75 | None | None |
| 70 | None | None |
| 65 | None | None |
| 60 | 3.0 | 1.0 |
| 55 | 2.0 | 0.0 |
| 50 | 1.0 | 0.0 |
| 45 | 1.0 | 0.0 |
| 40 | 2.0 | 0.0 |
| 35 | 2.0 | 0.0 |
| 30 | 2.0 | 0.0 |
| 25 | 1.0 | 1.0 |
| 20 | 1.0 | 3.0 |
| 15 | 1.0 | 0.0 |
| 10 | None | None |
| 5 | 1.0 | 2.0 |
| 0 | 0.0 | 4.0 |
| -5 | 0.0 | 5.0 |
| -10 | None | None |
| -15 | None | None |
| -20 | 0.0 | 1.0 |
| -25 | None | None |
| -30 | None | None |
| -35 | 0.0 | 1.0 |
| -40 | 0.0 | 1.0 |Number of cases
Numeric cyproterone-DILI-CAT score
