# Supplementary material for: A novel quantitative computer-assisted drug-induced liver injury causality assessment tool (DILI-CAT)
Source: PLoS One. 2022 Sep 29;17(9):e0271304. doi: 10.1371/journal.pone.0271304 (PMC9521919; doi:10.1371/journal.pone.0271304)
Supplement: S1 Table — (DOCX) [file pone.0271304.s003.docx]

**Supplemental Table 1: Examples for point allocation for latency throughout the first 100 days**

| **Latency (days)** | **Points** | | | |  | **Latency (days)** | **Points** | | | |
| --- | --- | --- | --- | --- | --- | --- | --- | --- | --- | --- |
|  | **Cyproterone** | **Cefazolin** | **AMX-CLA** | **PM** |  |  | **Cyproterone** | **Cefazolin** | **AMX-CLA** | **PM** |
| 1 | **-10** | **-10** | **-10** | 0 |  | 51 | 0 | -10 | 0 | 10 |
| 2 | **-10** | **-10** | -5 | 5 |  | 52 | 0 | -10 | 0 | 10 |
| 3 | **-10** | **-5** | -5 | 5 |  | 53 | 0 | -10 | 0 | 10 |
| 4 | **-10** | **-5** | 0 | 10 |  | 54 | 0 | -10 | 0 | 10 |
| 5 | **-10** | **-5** | 0 | 10 |  | 55 | 0 | -10 | 0 | 5 |
| 6 | **-10** | **-5** | 0 | 10 |  | 56 | 0 | -10 | 0 | 5 |
| 7 | **-10** | **-5** | 0 | 20 |  | 57 | 0 | -10 | 0 | 5 |
| 8 | **-10** | **-5** | 5 | 20 |  | 58 | 0 | -10 | 0 | 5 |
| 9 | **-10** | 10 | 10 | 20 |  | 59 | 0 | -10 | 0 | 5 |
| 10 | **-10** | 10 | 10 | 20 |  | 60 | 0 | -10 | 0 | 5 |
| 11 | **-10** | 10 | 10 | 20 |  | 61 | 0 | -10 | 0 | 5 |
| 12 | **-10** | 10 | 10 | 20 |  | 62 | 0 | -10 | 0 | 5 |
| 13 | **-10** | 10 | 10 | 20 |  | 63 | 5 | -10 | 0 | 5 |
| 14 | **-10** | 10 | 10 | 20 |  | 64 | 5 | -10 | -5 | 5 |
| 15 | **-10** | 20 | 10 | 20 |  | 65 | 5 | -10 | -5 | 5 |
| 16 | **-10** | 20 | 10 | 20 |  | 66 | 5 | -10 | -5 | 5 |
| 17 | **-10** | 20 | 20 | 20 |  | 67 | 5 | -10 | -5 | 5 |
| 18 | **-10** | 20 | 20 | 20 |  | 68 | 5 | -10 | -5 | 5 |
| 19 | -5 | 20 | 20 | 20 |  | 69 | 5 | -10 | -5 | 5 |
| 20 | -5 | 20 | 20 | 20 |  | 70 | 5 | -10 | -10 | 5 |
| 21 | -5 | 20 | 20 | 20 |  | 71 | 5 | -10 | -10 | 5 |
| 22 | -5 | 20 | 20 | 20 |  | 72 | 5 | -10 | -10 | 5 |
| 23 | -5 | 20 | 20 | 20 |  | 73 | 5 | -10 | -10 | 0 |
| 24 | -5 | 20 | 20 | 20 |  | 74 | 5 | -10 | -10 | 0 |
| 25 | -5 | 10 | 20 | 20 |  | 75 | 5 | -10 | -10 | 0 |
| 26 | -5 | 10 | 20 | 20 |  | 76 | 5 | -10 | -10 | 0 |
| 27 | -5 | 10 | 20 | 20 |  | 77 | 5 | -10 | -10 | 0 |
| 28 | -5 | 10 | 20 | 20 |  | 78 | 10 | -10 | -10 | 0 |
| 29 | -5 | 5 | 20 | 20 |  | 79 | 10 | -10 | -10 | 0 |
| 30 | -5 | 0 | 20 | 20 |  | 80 | 10 | -10 | -10 | 0 |
| 31 | -5 | -5 | 20 | 20 |  | 81 | 10 | -10 | -10 | 0 |
| 32 | -5 | -5 | 20 | 20 |  | 82 | 10 | -10 | -10 | 0 |
| 33 | 0 | -5 | 20 | 20 |  | 83 | 10 | -10 | -10 | 0 |
| 34 | 0 | -5 | 20 | 20 |  | 84 | 10 | -10 | -10 | 0 |
| 35 | 0 | -5 | 20 | 20 |  | 85 | 10 | -10 | -10 | 0 |
| 36 | 0 | -5 | 20 | 20 |  | 86 | 10 | -10 | -10 | 0 |
| 37 | 0 | -5 | 20 | 20 |  | 87 | 10 | -10 | -10 | 0 |
| 38 | 0 | -10 | 20 | 20 |  | 88 | 10 | -10 | -10 | 0 |
| 39 | 0 | -10 | 10 | 20 |  | 89 | 10 | -10 | -10 | 0 |
| 40 | 0 | -10 | 10 | 20 |  | 90 | 10 | -10 | -10 | 0 |
| 41 | 0 | -10 | 10 | 20 |  | 91 | 10 | -10 | -10 | 0 |
| 42 | 0 | -10 | 10 | 20 |  | 92 | 10 | -10 | -10 | 0 |
| 43 | 0 | -10 | 10 | 20 |  | 93 | 10 | -10 | -10 | 0 |
| 44 | 0 | -10 | 10 | 20 |  | 94 | 10 | -10 | -10 | 0 |
| 45 | 0 | -10 | 5 | 20 |  | 95 | 10 | -10 | -10 | 0 |
| 46 | 0 | -10 | 5 | 10 |  | 96 | 10 | -10 | -10 | 0 |
| 47 | 0 | -10 | 5 | 10 |  | 97 | 10 | -10 | -10 | 0 |
| 48 | 0 | -10 | 5 | 10 |  | 98 | 10 | -10 | -10 | 0 |
| 49 | 0 | -10 | 5 | 10 |  | 99 | 10 | -10 | -10 | 0 |
| 50 | 0 | -10 | 5 | 10 |  | 100 | 10 | -10 | -10 | 0 |

Bold numbers indicate respective outliers, gray background indicates numbers outside of range.

AMX/CLA, amoxicillin/clavulanate; PM, *polygonum multiflorum*.
