# Supplementary material for: A novel quantitative computer-assisted drug-induced liver injury causality assessment tool (DILI-CAT)
Source: PLoS One. 2022 Sep 29;17(9):e0271304. doi: 10.1371/journal.pone.0271304 (PMC9521919; doi:10.1371/journal.pone.0271304)
Supplement: S2 Table — S2a Table. Cyproterone DILI-CAT: This table shows the Cyproterone derived DILI-CAT-scoring algorithm comparing the cyproterone cases to the cases of the other drugs using the Cyproterone derived DILI-CAT; S2b Table. AMX/CLA DILI-CAT: This table shows the Amoxicilling/Clavulunaic (AMX/CLA) derived DILI-CAT-scoring algorithm comparing the AMX/CLA cases to the cases of the other drugs using the AMX/CLA derived DILI-CAT; S2c Table. Cefazolin DILI-CAT: This table shows the cefazolin derived DILI-CAT-scoring algorithm comparing the cefazolin cases to the cases of the other drugs using the cefazolin derived DILI-CAT; S2d Table. Polygonum multiflorum DILI-CAT: This table shows the Polygonum multiflorum derived DILI-CAT-scoring algorithm comparing the Polygonum multiflorum cases to the cases of the other drugs using the Polygonum multiflorum derived DILI-CAT cefazolin. (ZIP) [file pone.0271304.s004.zip › Sup Table 2a- Cyproterone DILI-CAT.docx]

**Supplemental Table 2a:** Cyproterone derived DILI-CAT-scoring algorithm comparing cyproterone to the other drugs using

|  |  | Clinical Features | | | Cyproterone derived DILI-CAT subscores | | | DiLI-CAT Scores | | |
| --- | --- | --- | --- | --- | --- | --- | --- | --- | --- | --- |
| Drug | Age | Latency | R-value | AST/ALT ratio | Latency Points | R-value Points | AST/ALT ratio points | Latency weighted | R-value weighted | AST/ALT ratio weighted |
| Cyproterone | 74 | 301 | 3.0 | 1.64 | 5 | 5 | 5 | 20 | 20 | 20 |
| Cyproterone | 66 | 425 | 21.6 | 0.78 | 0 | 5 | 20 | 25 | 30 | 45 |
| Cyproterone | 60 | 33 | 10.0 | 0.46 | 0 | 20 | 10 | 30 | 50 | 40 |
| Cyproterone | 72 | 151 | 1.0 | 1.75 | 20 | -5 | 0 | 35 | 10 | 15 |
| Cyproterone | 73 | 63 | 15.3 | 0.68 | 0 | 20 | 20 | 40 | 60 | 60 |
| Cyproterone | 66 | 96 | 7.2 | 0.58 | 10 | 10 | 10 | 40 | 40 | 40 |
| Cyproterone | 58 | 308 | 9.3 | 0.73 | 0 | 20 | 20 | 40 | 60 | 60 |
| Cyproterone | 83 | 64 | 11.7 | 0.36 | 5 | 20 | 10 | 40 | 55 | 45 |
| Cyproterone | 72 | 242 | 30.0 | 0.92 | 10 | 0 | 20 | 40 | 30 | 50 |
| Cyproterone | 80 | 122 | 7.1 | 2.13 | 20 | 10 | -5 | 45 | 35 | 20 |
| Cyproterone | 64 | 150 | 1.8 | 1.25 | 20 | -5 | 10 | 45 | 20 | 35 |
| Cyproterone | 64 | 100 | 17.8 | 0.66 | 10 | 20 | 10 | 50 | 60 | 50 |
| Cyproterone | 64 | 150 | 12.8 | 0.20 | 20 | 20 | -5 | 55 | 55 | 30 |
| Cyproterone | 77 | 246 | 9.8 | 0.90 | 10 | 20 | 20 | 60 | 70 | 70 |
| Cyproterone | 54 | 119 | 19.0 | 1.29 | 20 | 10 | 10 | 60 | 50 | 50 |
| Cyproterone | 74 | 125 | 27.8 | 0.70 | 20 | 0 | 20 | 60 | 40 | 60 |
| Cyproterone | 74 | 180 | 18.2 | 1.23 | 20 | 10 | 20 | 70 | 60 | 70 |
| Cyproterone | 58 | 123 | 15.0 | 0.68 | 20 | 20 | 20 | 80 | 80 | 80 |
| Cyproterone | 74 | 154 | 12.4 | 0.95 | 20 | 20 | 20 | 80 | 80 | 80 |
| Cyproterone | 74 | 149 | 13.4 | 0.70 | 20 | 20 | 20 | 80 | 80 | 80 |
| Cyproterone | 58 | 153 | 11.0 | 0.78 | 20 | 20 | 20 | 80 | 80 | 80 |
| Cyproterone | 75 | 240 | 11.5 | 0.79 | 20 | 20 | 20 | 80 | 80 | 80 |
| AMX_CLA |  | 10 | 0.2 | 1.87 | -10 | -10 | 0 | -30 | -30 | -20 |
| AMX_CLA |  | 6 | 1.6 | 0.21 | -10 | -5 | -5 | -30 | -25 | -25 |
| AMX_CLA |  | 14 | 0.5 | 1.53 | -10 | -10 | 5 | -25 | -25 | -10 |
| AMX_CLA |  | 26 | 0.6 | 1.82 | -5 | -10 | 0 | -20 | -25 | -15 |
| AMX_CLA |  | 4 | 1.6 | 0.56 | -10 | -5 | 10 | -15 | -10 | 5 |
| AMX_CLA |  | 11 | 0.6 | 0.95 | -10 | -10 | 20 | -10 | -10 | 20 |
| AMX_CLA |  | 28 | 1.9 | 0.38 | -5 | -5 | 10 | -5 | -5 | 10 |
| AMX_CLA |  | 7 | 1.0 | 0.69 | -10 | -5 | 20 | -5 | 0 | 25 |
| AMX_CLA |  | 8 | 1.3 | 0.75 | -10 | -5 | 20 | -5 | 0 | 25 |
| AMX_CLA |  | 26 | 1.9 | 0.37 | -5 | -5 | 10 | -5 | -5 | 10 |
| AMX_CLA |  | 19 | 2.3 | 0.62 | -10 | 5 | 10 | -5 | 10 | 15 |
| AMX_CLA |  | 50 | 1.0 | 0.50 | 0 | -10 | 10 | 0 | -10 | 10 |
| AMX_CLA |  | 44 | 0.8 | 0.55 | 0 | -10 | 10 | 0 | -10 | 10 |
| AMX_CLA |  | 44 | 0.7 | 0.54 | 0 | -10 | 10 | 0 | -10 | 10 |
| AMX_CLA |  | 38 | 0.7 | 0.43 | 0 | -10 | 10 | 0 | -10 | 10 |
| AMX_CLA |  | 24 | 0.7 | 0.87 | -5 | -10 | 20 | 0 | -5 | 25 |
| AMX_CLA |  | 24 | 0.4 | 1.11 | -5 | -10 | 20 | 0 | -5 | 25 |
| AMX_CLA |  | 22 | 3.1 | 0.58 | -5 | 5 | 10 | 5 | 15 | 20 |
| AMX_CLA |  | 27 | 1.8 | 0.94 | -5 | -5 | 20 | 5 | 5 | 30 |
| AMX_CLA |  | 37 | 1.1 | 0.65 | 0 | -5 | 10 | 5 | 0 | 15 |
| AMX_CLA |  | 8 | 3.1 | 0.80 | -10 | 5 | 20 | 5 | 20 | 35 |
| AMX_CLA |  | 26 | 1.1 | 0.81 | -5 | -5 | 20 | 5 | 5 | 30 |
| AMX_CLA |  | 20 | 1.2 | 0.87 | -5 | -5 | 20 | 5 | 5 | 30 |
| AMX_CLA |  | 38 | 1.0 | 0.49 | 0 | -5 | 10 | 5 | 0 | 15 |
| AMX_CLA |  | 26 | 3.2 | 0.32 | -5 | 5 | 10 | 5 | 15 | 20 |
| AMX_CLA |  | 25 | 4.5 | 0.45 | -5 | 5 | 10 | 5 | 15 | 20 |
| AMX_CLA |  | 17 | 2.9 | 0.70 | -10 | 5 | 20 | 5 | 20 | 35 |
| AMX_CLA |  | 35 | 0.8 | 0.91 | 0 | -10 | 20 | 10 | 0 | 30 |
| AMX_CLA |  | 23 | 2.1 | 0.98 | -5 | 0 | 20 | 10 | 15 | 35 |
| AMX_CLA |  | 20 | 7.8 | 0.55 | -5 | 10 | 10 | 10 | 25 | 25 |
| AMX_CLA |  | 63 | 0.6 | 1.14 | 0 | -10 | 20 | 10 | 0 | 30 |
| AMX_CLA |  | 41 | 2.8 | 0.57 | 0 | 5 | 10 | 15 | 20 | 25 |
| AMX_CLA |  | 54 | 6.1 | 0.50 | 0 | 10 | 10 | 20 | 30 | 30 |
| AMX_CLA |  | 44 | 13.9 | 0.33 | 0 | 20 | 10 | 30 | 50 | 40 |
| AMX_CLA |  | 50 | 12.0 | 0.85 | 0 | 20 | 20 | 40 | 60 | 60 |
| Cefazolin |  | 6 | 1.5 | 0.56 | -10 | -5 | 10 | -15 | -10 | 5 |
| Cefazolin |  | 15 | 1.8 | 0.38 | -10 | -5 | 10 | -15 | -10 | 5 |
| Cefazolin |  | 7 | 1.0 | 0.49 | -10 | -5 | 10 | -15 | -10 | 5 |
| Cefazolin |  | 7 | 1.2 | 0.28 | -10 | -5 | 10 | -15 | -10 | 5 |
| Cefazolin |  | 21 | 6.1 | 0.19 | -5 | 10 | -10 | -10 | 5 | -15 |
| Cefazolin |  | 29 | 0.8 | 0.39 | -5 | -10 | 10 | -10 | -15 | 5 |
| Cefazolin |  | 18 | 0.5 | 1.23 | -10 | -10 | 20 | -10 | -10 | 20 |
| Cefazolin |  | 18 | 3.4 | 0.51 | -10 | 5 | 10 | -5 | 10 | 15 |
| Cefazolin |  | 27 | 0.9 | 0.99 | -5 | -10 | 20 | 0 | -5 | 25 |
| Cefazolin |  | 20 | 2.2 | 0.38 | -5 | 5 | 10 | 5 | 15 | 20 |
| Cefazolin |  | 20 | 1.1 | 0.97 | -5 | -5 | 20 | 5 | 5 | 30 |
| Cefazolin |  | 20 | 2.9 | 0.46 | -5 | 5 | 10 | 5 | 15 | 20 |
| Cefazolin |  | 20 | 1.3 | 0.74 | -5 | -5 | 20 | 5 | 5 | 30 |
| Cefazolin |  | 24 | 4.5 | 0.39 | -5 | 5 | 10 | 5 | 15 | 20 |
| Cefazolin |  | 29 | 1.1 | 0.81 | -5 | -5 | 20 | 5 | 5 | 30 |
| Cefazolin |  | 28 | 1.6 | 0.66 | -5 | -5 | 20 | 5 | 5 | 30 |
| Cefazolin |  | 26 | 2.8 | 0.36 | -5 | 5 | 10 | 5 | 15 | 20 |
| Cefazolin |  | 24 | 3.4 | 0.37 | -5 | 5 | 10 | 5 | 15 | 20 |
| Cefazolin |  | 23 | 10.6 | 0.33 | -5 | 20 | 10 | 20 | 45 | 35 |
| Polygonum Multiforum |  | 15 | 2.8 | 2.52 | -10 | 5 | -10 | -25 | -10 | -25 |
| Polygonum Multiforum |  | 1 | 20.7 | 0.52 | -10 | 5 | 10 | -5 | 10 | 15 |
| Polygonum Multiforum |  | 7 | 26.3 | 0.91 | -10 | 0 | 20 | 0 | 10 | 30 |
| Polygonum Multiforum |  | 7 | 5.5 | 0.42 | -10 | 10 | 10 | 0 | 20 | 20 |
| Polygonum Multiforum |  | 20 | 7.0 | 0.58 | -5 | 10 | 10 | 10 | 25 | 25 |
| Polygonum Multiforum |  | 7 | 9.1 | 0.52 | -10 | 20 | 10 | 10 | 40 | 30 |
| Polygonum Multiforum |  | 4 | 6.0 | 0.73 | -10 | 10 | 20 | 10 | 30 | 40 |
| Polygonum Multiforum |  | 10 | 11.9 | 0.42 | -10 | 20 | 10 | 10 | 40 | 30 |
| Polygonum Multiforum |  | 14 | 13.5 | 0.42 | -10 | 20 | 10 | 10 | 40 | 30 |
| Polygonum Multiforum |  | 28 | 7.8 | 0.97 | -5 | 10 | 20 | 20 | 35 | 45 |
| Polygonum Multiforum |  | 52 | 7.9 | 0.35 | 0 | 10 | 10 | 20 | 30 | 30 |
| Polygonum Multiforum |  | 50 | 5.5 | 0.26 | 0 | 10 | 10 | 20 | 30 | 30 |
| Polygonum Multiforum |  | 23 | 10.9 | 0.44 | -5 | 20 | 10 | 20 | 45 | 35 |
| Polygonum Multiforum |  | 29 | 13.0 | 0.50 | -5 | 20 | 10 | 20 | 45 | 35 |
| Polygonum Multiforum |  | 43 | 18.6 | 0.60 | 0 | 10 | 10 | 20 | 30 | 30 |
| Polygonum Multiforum |  | 1 | 16.8 | 0.74 | -10 | 20 | 20 | 20 | 50 | 50 |
| Polygonum Multiforum |  | 67 | 12.1 | 0.32 | 5 | 20 | 10 | 40 | 55 | 45 |
| Polygonum Multiforum |  | 120 | 7.6 | 0.56 | 20 | 10 | 10 | 60 | 50 | 50 |

**Gray boxes indicate the respective median values in weighting DILI-CAT score, the U-value derived from the greatest difference in Mann-Whitney non-parametric tests dictates which weighting DILI-CAT score to use**
