# Supplementary material for: A novel quantitative computer-assisted drug-induced liver injury causality assessment tool (DILI-CAT)
Source: PLoS One. 2022 Sep 29;17(9):e0271304. doi: 10.1371/journal.pone.0271304 (PMC9521919; doi:10.1371/journal.pone.0271304)
Supplement: S1 Appendix — (DOCX) [file pone.0271304.s005.docx]

**Supplement Appendix 1**

**Outlier definition:**

Because we recognized that some cases would include data that way outside of the pre-specified range (“outliers”), we developed a standard definition of outliers above and below the interquartile range (IQR) is IQR_high_+IQR*1.5 for values above the IQR and IQR_low_-IQR*1.5 for values below the IQR.

This standard definition does not work for the DILI parameters when the following occur:

1.) When the latency would be a negative number of days,

2.) When the R-value or AST/ALT are ratios would be negative.

Therefore, we defined outliers in this study as IQR_high_+IQR*1.5 for values above the IQR, but IQR_low_-IQR*0.75 for values below the IQR.
