# Supplementary material for: A novel quantitative computer-assisted drug-induced liver injury causality assessment tool (DILI-CAT)
Source: PLoS One. 2022 Sep 29;17(9):e0271304. doi: 10.1371/journal.pone.0271304 (PMC9521919; doi:10.1371/journal.pone.0271304)
Supplement: S2 Appendix — (DOCX) [file pone.0271304.s006.docx]

**Supplemental Material, Appendix 2**

**Point allocation for competing causes and the lack thereof (-25 to 25 points)**

For this project we had a few “a priori” considerations:

1. If all alternative causes were truly excluded, the score should, at minimum, result in a score of possible, even when the respective drug was not known to be hepatotoxic, and therefore no phenotype of the drug is known.

2. Even if an alternative cause is identified but a case’s injury pattern completely fits the expected phenotype, the resulting score should not exclude drug-induced liver injury (DILI) completely.

Thus, if a definite alternative cause of abnormal liver tests was identified, 25 points were to be deducted. If no alternative cause was positively identified, completeness of exclusion of alternative causes is important, since DILI is a diagnosis of exclusion. We arbitrarily allocated 25 points if all relevant competing causes were excluded under the premise that a case with suspected DILI to a drug with no previous report of hepatotoxicity should be adjudicated as possible DILI. Depending on the injury pattern, hepatocellular vs. cholestatic, different diseases needed to be excluded. In the case of a mixed injury pattern, both causes of hepatocellular and cholestatic injury needed to be excluded. If not all compatible diseases were excluded, various points were deducted from the 25 points depending on a weighted importance of a competing cause to be excluded.

Diseases differ in their importance to be excluded. Thus, a relative exclusionary importance of various causes of liver disease was developed (Table “**Relative importance of competing causes**“ below). The resulting point deduction from 25 potential points was derived from the respective score for the exclusion of the formular 25 minus 25 points multiplied by the relative importance of missing excluded diagnosis from 25 points. If the sum of relative importance of missing values excluded competing diagnosis was between 1 and <2, a relative importance value of 1 was used. If relative importance of missing values was ≥2 a relative value of 1.4 was used (see examples for details).

Examples:

- If the sum of relative importance of missing values was 0.3, a total of 7.5 points were deducted from 25 points, resulting in a net value of 17.5 points to be added to the score.
- If the sum of relative importance of missing values was 0.7, a total of 17.5 points were deducted from 25 points, resulting in a net value of 7.5 points to be added to the score.
- If the sum of relative importance of missing values was between 1 and <2, a relative importance value of 1 was used, resulting in 25 points to be deducted from 25 points, resulting in a net value of no point to be added to the score.
- If the sum of relative importance of missing values was ≥2, 35 points were to be deducted from the 25 points, resulting in a net value of 10 points to be subtracted from the total score.

**Table on** “**Relative importance of competing causes”**

|  | **Relative importance** |
| --- | --- |
| **Competing causes for a hepatocellular injury pattern** | |
| a.) Hepatitis viruses A to E |  |
| HAV (IgM) | 1 |
| HBV (HBcIgM) | 1 |
| HCV (anti-HCV or HCV-RNA positive) | 1 |
| HDV (IgM) (only required if HBsAg or anti-HBcIgM is positive) | 1 |
| HEV (IgM)** | 0.2 |
| b.) Alcohol abuse (AST ULN>ALT ULN × 1.5) and max AST <500 | 0.8 |
| c.) Herpesvirus: EBV, CMV or HSV or VZV | 0.3 |
| d.) Autoimmune hepatitis based on AIH Score ≥ 6* | 0.8 |
| e.) Ischemic hepatitis (evidence for hypotension) | 0.8 |
| f.) Imaging – US, CT, or MRI – biliary dilation finding only | 0.7 |
| g.) Gallstones with clinic of passage (hx of acute pain) | 0.8 |
| **Competing causes for a cholestatic injury pattern** | |
| a.) AMA (anti-mitochondrial antibodies) | 0.8 |
| b.) IgG4 levels | 0.5 |
| c.) Imaging – US, CT, MRCP, or ERCP | 1 |

* Based on ANA/SMA 1:40 or 1:80, IgG >1.0 or >1.1, histology compatible, exclusion of viral hepatitis.

** HEV IgM may deserve a higher relative value in Europe versus the United States, where the incidence of acute hepatitis E seems rarer.

AIH, autoimmune hepatitis; ALT, alanine aminotransferase; AST, aspartate aminotransferase; CMV, cytomegalovirus; CT, computerized tomography; EBV, Epstein-Barr virus; ERCP, endoscopic retrograde cholangiopancreatography; HSV, herpes simplex virus; Ig, immunoglobulin; MRCP, magnetic resonance cholangiopancreatography; ULN, upper limit of normal; US, ultrasonography; VZV, varicella zoster virus.
