# Supplementary material for: A novel quantitative computer-assisted drug-induced liver injury causality assessment tool (DILI-CAT)
Source: PLoS One. 2022 Sep 29;17(9):e0271304. doi: 10.1371/journal.pone.0271304 (PMC9521919; doi:10.1371/journal.pone.0271304)
Supplement: S3 Appendix — (DOCX) [file pone.0271304.s007.docx]

**Supplemental Material Appendix 3**

**Hepatotoxicity potential (0-20 points)**

Drugs have different propensities for causing drug-induced liver injury (DILI). *LiverTox* (available at Pubmed.ncbi.nlm.nih.gov) provides an estimate of the propensity that a certain drug will cause DILI and, thus, we used this scoring system. This system can be used for the following point allocations:

Drugs with ≥ 50 cases reported in the literature = 20 points

Drugs with ≥ 12 but < 50 cases reported in the literature = 15 points

Drugs with ≥ 4 but <12 cases reported in the literature = 10 points

Drugs with 1 to 3 cases reported in the literature = 10 points

Drugs with no cases reported in the literature = 0 points
