# Supplementary material for: A novel quantitative computer-assisted drug-induced liver injury causality assessment tool (DILI-CAT)
Source: PLoS One. 2022 Sep 29;17(9):e0271304. doi: 10.1371/journal.pone.0271304 (PMC9521919; doi:10.1371/journal.pone.0271304)
Supplement: S4 Appendix — (DOCX) [file pone.0271304.s008.docx]

**Supplemental Material Appendix 4**

The summation of points in the three categories (DILI-CAT phenotype, hepatotoxicity, and competing causes) theoretical ranges from -65 [drug with no history of hepatotoxicity (0 points), completely not fitting the signature (-40 points) and positive alternative cause (-25 points)] to 125 [drug known to be frequently hepatotoxic (20 points), complete fit with DILI-CAT phenotype (80 points), and all alternative causes excluded (25 points)].

The sum score would be graded for likelihood where 95 points or higher would be “definite DILI”, 75 to 95 points would be “highly likely DILI,” 50 to 75 points would be “probable DILI,” 25 to 50 points would be “possible DILI,” and less than 25 points would be “unlikely DILI.”
